# ICU and TIPS, our experience in a community hospital in spain

**DOI:** 10.1186/2197-425X-3-S1-A696

**Published:** 2015-10-01

**Authors:** AM Leal Micharet, A Martín Vivas, M Yagüe Huertas, J Ferrero Calleja, A Hernández Tejedor, R Ruiz de Luna, M Sigcha Sigcha, N de la Calle Pedrosa, Á Gabán Díez, AI González Jiménez, I Temprano Gómez, A Algora Weber

**Affiliations:** Intensive Care Unit, Hospital Universitario Fundación Alcorcón, Alcorcón, Spain

## Introduction

Transjugular intrahepatic portosystemic shunt (TIPS) is a non selective portosystemic derivation created by endovascular techniques. A transhepatic comunication between one of the hepatic veins and a branch of portal vein is created using a needle system. Intrahepatic trajectory remains open by placing a metallic stent. TIPS is an effective method to relieve the portal system pressure. Portal hipertension is one of the major consecuences in cirrhosis, and its main complications are variceal bleeding, refractary ascites (RA) and hydrothorax. Being these ones the principal indications for TIPS. The complications related to the TIPS procedure are these related to the technique: access (capsular laceration, intraperitoneal bleeding, hepatic infarction, fistula, hemobilia) or stent (thrombosis, occlusion, migration, sepsis); or related to portosistemic shunt (hepatic encephalopaty (HE), hemodynamic failure, sepsis).

## Objectives

The aim of this study is to analize the epidemiologic characteristics of the patients that required TIPS and were admitted in an Intensve Care Unit (ICU), and its indications, complications and evolution after the procedure.

## Methods

We realized a descriptive retrospective study of the epidemiologic, clinical and evolutive characteristics of the patients who required a TIPS and were admitted in our ICU (polivalent ICU in a community hospital), during 2000-2015. We assessed the prognosis of chronic liver disease by Child-Pugh score (CP) and Model for End-Stage Liver Disease (MELD).

## Results

27 patients were included, 81% men, median age 53years-old (range 28-79). The etiologies of the liver disease were alocholic (66.7%), HCV (7.4%), alcoholic and HCV (3.7%), HBV (3.7%), alcoholic and HBV (3.7%), cryptogenic (11.1%), previously unknown (3.7%). The liver disease was staged by CP: A 14.8%, B 48,2%, C 37% and MELD: < 9=3.7%, 10-19=51.8%, 20-29=37.1%, >30=3,7%, uncertain in 3.7%. 62% of TIPS were urgent. Indications were gastrointestinal bleeding (GIB) (74,1%), RA (22.2%), hydrothorax (3.7%). Pressure gradement was measured (pre/post TIPS) in 77.7%, mean pre 19 ± 5 mmHg/post 8 ± 4 mmHg. Bleeding related to the technique was the unique acute complication, found in 14%. Subacute complication developed in 48.15% (hemodynamic failure 7.4%, hepatic failure 7.4%, HE 11.1%, stent thrombosis 22.2%). 40.7% of TIPS failed, because of bleeding 22.2%, HE 11.1%, hydrothorax 3.7%, ascites 18.1% Besides, renal failure was developed in 22.2%. ICU mortality was 33.3%.

## Conclusions

The typical patient that needs TIPS and admission in ICU is a mild-age man, diagnosed of alcoholic liver disease, staged in CP B, mean MELD 19, that develops a GIB and needs an urgent TIPS. The technique itself is related to a significant number of acute and subacute complications. The risk of death is moderately elevated but lower tan estimated by scores.
